# Seroprevalence of Poliovirus Antibodies in the United States Population, 2009–2010

**DOI:** 10.1186/s12889-016-3386-1

**Published:** 2016-08-05

**Authors:** Gregory S. Wallace, Aaron T. Curns, William C. Weldon, M. Steven Oberste

**Affiliations:** Division of Viral Diseases, Center for Disease Control and Prevention, Atlanta, Georgia

**Keywords:** Polio, Poliovirus, NHANES, Seroepidemiologic studies, Antibodies

## Abstract

**Background:**

Polio is eliminated in the United States, with the last indigenous transmission occurring in 1979. However, global eradication of polio has not yet been completed, so importation of poliovirus into the U.S. is still possible. Specimens from the 2009-10 National Health and Nutrition Examination Survey (NHANES) were analyzed to evaluate population seroprevalence and assess overall risk from a poliovirus importation.

**Methods:**

We evaluated prevalence of serum antibodies to all three poliovirus types using the National Health and Nutrition Examination Survey during 2009–2010.

**Results:**

The overall seroprevalence to poliovirus was 93.9 % for type 1, 97.0 % for type 2, and 83.1 % for type 3. Seroprevalence was higher for type 2 compared to the other types (*p* < 0.001) and lower for type 3 compared to the other types (*p* < 0.001). There was a tendency for higher seroprevalence in the younger age groups, but this varied by serotype.

**Conclusions:**

Seroprevalence was high (83.1 %–97.0 %) for all three types of poliovirus in the US population during 2009–2010. While there were observed differences by serotype with type 2 having the highest seroprevalence and type 3 having the lowest, consistent with previous observations, no large immunity gaps to poliovirus suggesting an imminent substantial population risk from a poliovirus importation were observed at a population level.

## Background

Pakistan and Afghanistan remain the only two countries where wild poliovirus (WPV) transmission has never been interrupted [1–3]. While the last cases of indigenously acquired WPV in the United States (U.S.) occurred in 1979, the last WPV case in a U.S. resident traveling abroad occurred in 1986, and the last WPV imported case occurred in 1993. Due to continued WPV transmission in a few remaining areas of the world, CDC has provided interim vaccination guidance for travel to and from countries affected by wild poliovirus [4].

**Fig. 1:**
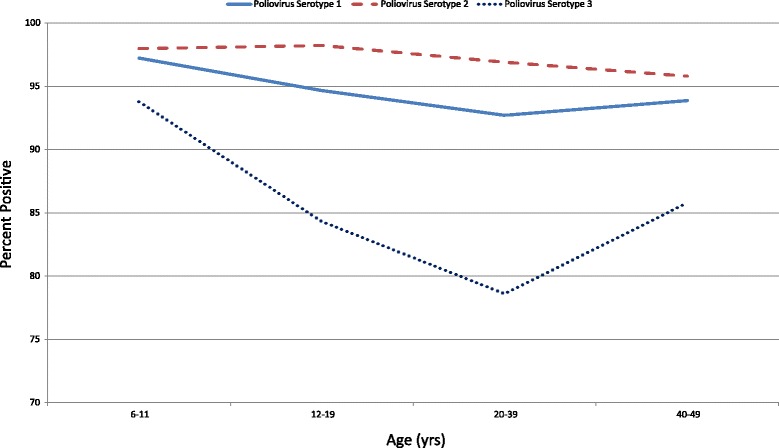
Seroprevalence of Poliovirus Antibodies by Birth Cohort

**Fig. 2:**
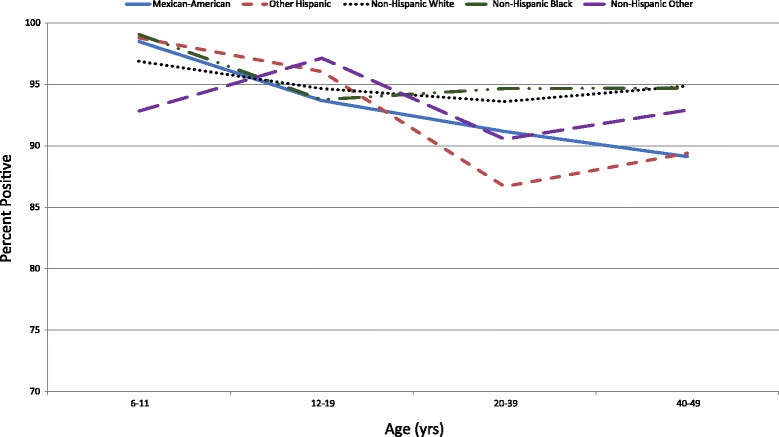
Seroprevalence of Poliovirus Antibodies by Birth Cohort & Race/Ethnicity for Serotype 1

**Fig. 3:**
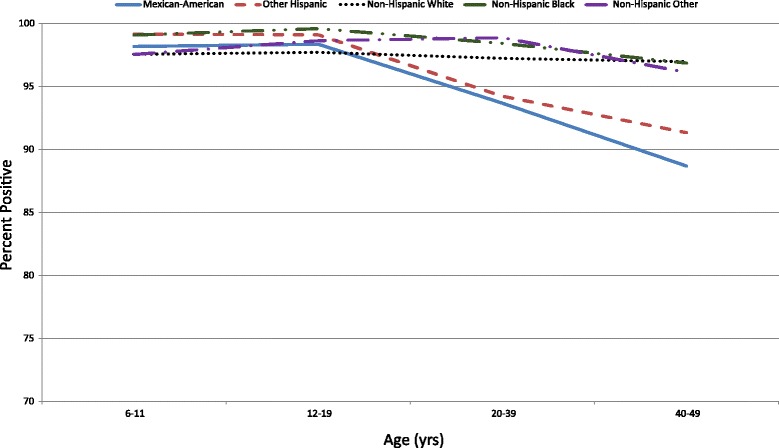
Seroprevalence of Poliovirus Antibodies by Birth Cohort & Race/Ethnicity for Serotype 2

**Fig. 4:**
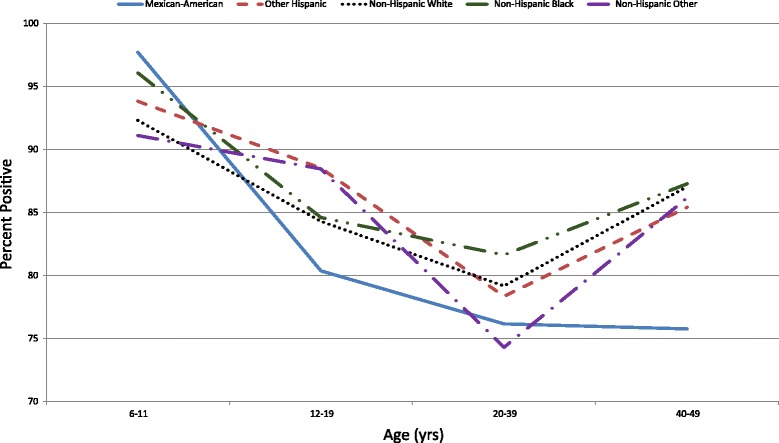
Seroprevalence of Poliovirus Antibodies by Birth Cohort & Race/Ethnicity for Serotype 3

Additionally, circulating vaccine-derived poliovirus (cVDPV) must also be eliminated before polio eradication is achieved [5]. Since 2000, the United States has exclusively used inactivated poliovirus vaccine (IPV), in order to prevent vaccine-associated paralytic poliovirus cases (VAPP) which averaged 8–10 cases per year in the U.S. when oral poliovirus vaccine (OPV) was routinely recommended [6]. No systematic serosurveys for poliovirus antibodies have been conducted in the U.S. since the return to an all-IPV recommendation [7, 8]. This study describes the findings of a serosurvey conducted using the 2009-10 National Health and Nutrition Examination Survey (NHANES).

## Methods

NHANES is conducted by the National Center for Health Statistics (NCHS), Centers for Disease Control and Prevention (CDC), to assess the health and nutritional status of the US population [9]. The survey uses a stratified, multistage, probability-cluster design, to provide a sample representative of the civilian non-institutionalized US population. Survey participants undergo household interviews and standardized physical examinations, and provide biological samples for testing. Non-Hispanic blacks and Hispanics were over-sampled to provide stable estimates for these groups. Informed consent was obtained from all participants, and the Institutional Review Board of NCHS, CDC approved the protocol.

Seroprevalence for antibodies against poliovirus serotypes 1-3 was compared by demographic characteristics for 4806 individuals aged 6 to 49 years who participated in the 2009–2010 NHANES. The NHANES sample is designed to provide population-based estimates that are nationally representative of the US population [10]. Seroprevalence was compared by gender, age group (6–11, 12–19, 20–39, and 40–49 years), race/ethnicity (Mexican-American, Other Hispanic, Non-Hispanic White, Non-Hispanic Black, Other Race – Including Multi-Racial), and country of birth (United States or Other).

The data were analyzed using SUDAAN 11.0 (Research Triangle Institute, Research Triangle Park, NC) and SAS 9.3 (SAS Institute, Cary NC). t-tests were used to evaluate differences in seroprevalence by demographic characteristics and serotype. *P*-values <0.05 were considered statistically significant and were not adjusted for multiple comparisons. All statistical analyses and 95 % Clopper-Pearson confidence intervals for the seroprevalence point estimates accounted for the NHANES complex survey design [10].

Serum samples from NHANES participants aged 6–49 years during 2009–2010 were tested for poliovirus-specific antibodies. Samples were tested using a standard microneutralization assay for antibodies to poliovirus types 1, 2, and 3, according to established protocols at the Global Polio Specialized Laboratory, Centers for Disease Control and Prevention (CDC) [11]. Briefly, 80-100 CCID_50_ (50 % cell culture infectious dose) of each poliovirus serotype and two-fold serial dilutions of serum were separately combined and pre-incubated at 35 °C for 3 h before addition of HEp-2(C) cells (human cervix carcinoma cell line). After incubation for 5 days at 35 °C and 5 % CO_2_, plates were stained with crystal violet and cell viability measured by optical density in a plate spectrophotometer. Each specimen was run in triplicate, with parallel specimens from one study subject tested in the same assay run, and the neutralization titers estimated by the Spearman-Kärber method [12] and reported as the reciprocal of the calculated 50 % endpoint. Each run contained multiple replicates of a reference antiserum pool to monitor performance variation. A serum sample was considered positive if the 50 % endpoint titer was ≥1:8 dilution.

## Results

### Response rates

Of 6981 persons aged 6–49 years sampled for NHANES 2009–2010, 5785 (83 %) were interviewed and 5652 (81 %) were examined. Of those examined, 4806 (85 %) had serum samples available for poliovirus antibody testing. The proportion of examined participants with tested samples varied by age (6–11 years, 74 %; 12–19 years, 85 %; 20–39 years, 89 %; 40–49 years, 91 %; *p* < 0.001), race/Hispanic origin (Mexican American, 91 %; Other Hispanic, 76 %; non-Hispanic white, 85 %; non-Hispanic black, 82 %; non-Hispanic Other/Multi-racial, 88 %; *p* < 0.001), and birthplace (US, 84 %; non-US, 87 %; *p* < 0.05), but not by gender.

### Overall seroprevalence of poliovirus antibody for types 1, 2, and 3

During 2009–2010, among the US population aged 6–49 years, overall poliovirus seroprevalence for types 1, 2, and 3 was 93.9 % (95 % CI: 92.5 %–95.0 %), 97.0 % (95 % CI: 95.9 %–97.9 %) and 83.1 % (95 % CI: 80.7 %–85.4 %), respectively. Seroprevalence was higher for type 2 compared to type 1 and type 3 (*p* < 0.001 for each comparison) and seroprevalence for type 1 was also higher than type 3 (*p* <0.001). Less than 1 % of those tested were negative to all 3 serotypes.

### Seroprevalence of poliovirus type 1 antibody

Poliovirus seroprevalence did not differ by gender but was higher among those aged 6–11 years compared to those aged 20–39 and 40–49 years (*p* < 0.05 for each comparison, Table 1, Fig. 1). When stratified by gender, those aged 6–11 years had a higher seroprevalence than those aged 20–39 for both males and females (*p* < 0.05 and *p* < 0.001, respectively). No other differences between other age groups were observed for either gender. Within each age group and overall, no differences by gender were observed. The U.S. born group had poliovirus seroprevalence levels higher than the non-U.S. born group (*p* < 0.05).

**Table 1:** Seroprevalence in the United States to Poliovirus by Serotype and Demographics

	Poliovirus Serotype 1	Poliovirus Serotype 2	Poliovirus Serotype 3
	Percent positive (+/- 95 % Confidence Interval)	Percent positive (+/- 95 % Confidence Interval)	Percent positive (+/- 95 % Confidence Interval)
Gender			
Female	93.4 (91.7–94.9)	97.3 (96.1–98.2)	83.5 (80.4–86.4)
Male	94.3 (92.4–95.8)	96.6 (95.1–97.8)	82.7 (80.4–84.9)
Age Group (yrs)			
6–11	97.2 (94.7–98.8)	98.0 (96.4–99.0)	93.8 (91.8–95.4)
12–19	94.7 (92.0–96.6)	98.2 (96.6–99.2)	84.3 (81.0–87.2)
20–39	92.7 (90.0–94.2)	96.9 (95.2–98.2)	78.6 (74.6–82.2)
40–49	93.9 (91.6–95.7)	95.8 (93.8–97.3)	85.8 (82.3–88.8)
Gender and Age			
Female			
6–11	97.5 (94.8–99.0)	98.8 (96.3–99.8)	93.6 (90.5–95.9)
12–19	94.7 (90.7–97.3)	98.4 (96.6–99.4)	82.8 (78.0–86.2)
20–39	91.9 (89.5–93.9)	97.0 (94.3–98.7)	78.6 (73.9–82.9)
40–49	93.6 (89.9–96.3)	96.6 (94.3–98.1)	88.5 (84.6–91.7)
Male			
6–11	97.0 (92.6–99.2)	97.2 (94.8–98.7)	94.0 (91.2–96.1)
12–19	94.7 (90.0–97.6)	98.0 (95.7–99.3)	85.8 (80.6–90.0)
20–39	93.5 (91.0–95.5)	96.8 (95.4–97.9)	78.6 (74.1–82.6)
40–49	94.1 (91.5–96.1)	95.0 (91.4–97.4)	82.9 (78.4–86.8)
Race/Ethnicity			
Mexican-American	92.4 (89.6–94.6)	94.3 (92.4–95.9)	80.3 (77.0–82.5)
Other Hispanic	90.1 (84.5–94.2)	94.9 (91.3–97.4)	83.2 (78.6–87.2)
Non-Hispanic White	94.5 (92.6–96.0)	97.3 (95.9–98.3)	83.6 (80.9–86.0)
Non-Hispanic Black	95.0 (93.0–96.6)	98.4 (97.3–99.2)	85.2 (80.8–89.0)
Non-Hispanic Other	92.5 (86.8–96.2)	98.1 (94.8–99.6)	81.3 (70.8–89.2)
Age & Race/Ethnicity			
6–11			
Mexican-American	98.5 (96.4–99.5)	98.2 (96.0–99.4)	97.7 (94.7–99.3)
Other Hispanic	98.8 (93.5–100.0)	99.2 (94.7–100.0)	93.8 (87.0–97.7)
Non-Hispanic White	96.9 (93.1–98.9)	97.6 (94.9–99.1)	92.3 (88.5–95.2)
Non-Hispanic Black	99.1 (96.0–99.9)	99.1 (96.3–99.9)	96.1 (90.6–98.8)
Non-Hispanic Other	92.8 (83.1–97.9)	97.6 (87.6–99.9)	91.1 (81.0–96.9)
12–19			
Mexican-American	93.7 (90.5–96.1)	98.4 (96.3–99.4)	80.4 (74.5–85.4)
Other Hispanic	96.1 (90.2–98.9)	99.1 (94.7–100.0)	88.5 (81.0–93.8)
Non-Hispanic White	94.7 (89.5–97.8)	97.7 (95.1–99.2)	84.3 (79.3–88.5)
Non-Hispanic Black	93.8 (89.4–96.7)	99.6 (97.7–100.0)	84.6 (79.3–89.0)
Non-Hispanic Other	97.1 (90.7–99.6)	98.6 (93.0–100.0)	88.5 (79.3–94.6)
20–39			
Mexican-American	91.2 (86.1–94.9)	93.7 (90.2–96.2)	76.2 (71.5–80.4)
Other Hispanic	86.7 (78.5–92.6)	94.2 (89.8–97.1)	78.4 (70.5–84.9)
Non-Hispanic White	93.6 (91.2–95.5)	97.2 (95.3–98.5)	79.2 (75.1–82.9)
Non-Hispanic Black	94.7 (91.5–96.9)	98.4 (96.4–99.5)	81.6 (75.8–86.6)
Non-Hispanic Other	90.5 (82.4–95.8)	98.9 (92.4–100.0)	74.3 (54.9–88.6)
40–49			
Mexican-American	89.1 (83.9–93.1)	88.7 (80.9–94.1)	75.8 (69.5–81.3)
Other Hispanic	89.4 81.6–94.7)	91.3 (82.4–96.7)	85.4 (72.5–93.8)
Non-Hispanic White	94.9 (91.7–97.1)	97.0 (94.3–98.6)	87.0 (82.1–91.0)
Non-Hispanic Black	94.7 (90.0–97.6)	96.9 (92.1–99.2)	87.3 (78.7–93.8)
Non-Hispanic Other	92.9 (75.0–99.2)	96.1 (87.2–99.5)	86.2 (68.9–95.9)
U.S. Born			
Yes	94.5 (93.2–95.6)	97.5 (96.3–98.4)	83.9 (81.6–86.1)
No	90.8 (87.4–93.6)	94.6 (92.7–96.1)	79.4 (72.9–84.9)
Total	93.9 (92.5–95.0)	97.0 (95.9–97.9)	83.1 (80.7–85.4)

Non-Hispanic Blacks had higher seroprevalence compared to Mexican-Americans and Other Hispanics (*p* < 0.05 for each comparison). When stratified by age, no differences were observed by race/ethnicity within the 6–11 or 12–19 year age group (Fig. 2). Within the 20–39 year age group, Non-Hispanic Blacks had a higher seroprevalence than Other Hispanics (*p* < 0.05). Within the 40–49 year age group, Non-Hispanic Blacks had a higher seroprevalence than Mexican-Americans and Non-Hispanic Whites had a higher seroprevalence than Mexican-Americans and Other Hispanics (*p* < 0.05).

Among Mexican-Americans, those aged 6–11 years had a higher seroprevalence than those aged 12–19, 20–39, and 40–49 years (*p* < 0.05, *p* < 0.05, and *p* < 0.001, respectively). Those aged 12–19 years had a higher seroprevalence than those aged 40–49 years (*p* < 0.05).

Similarly, among Other Hispanics, those aged 6–11 years had a higher seroprevalence than those aged 20–39 and 40–49 years (*p* < 0.05 for each comparison), and those aged 12–19 years had a higher seroprevalence than those aged 40–49 years (*p* < 0.05).

Among Non-Hispanic Whites, those aged 6–11 years had a higher seroprevalence than those aged 20–39 years (*p* < 0.05). Among Non-Hispanic Blacks, those aged 6–11 years had a higher seroprevalence than those aged 12–19, 20–39, and 40–49 years (*p* < 0.05 for each comparison). No statistically significant differences were observed by age group within Non-Hispanic Others.

### Seroprevalence of poliovirus type 2 antibody

Poliovirus seroprevalence did not differ by gender but was higher among those aged 6–11 years compared to those aged 40–49 years (*p* < 0.05, Table 1, Fig. 1). Those aged 12–19 years had a higher seroprevalence than those aged 40–49 years (*p* < 0.05). When stratified by gender, those aged 6–11 years had a higher seroprevalence than those aged 40–49 years for females (*p* < 0.05) and those aged 12–19 years had a higher seroprevalence than those aged 40–49 years for males (*p* < 0.05). No other differences between other age groups were observed for either gender. Within each age group, no differences by gender were observed. The U.S. born group had poliovirus seroprevalence levels higher than the non-U.S. born group (*p* < 0.05).

Non-Hispanic Blacks had higher seroprevalence compared to Mexican-Americans and Other Hispanics (*p* < 0.001, *p* < 0.05, respectively). Non-Hispanic Whites and Non-Hispanic Other also had a higher seroprevalence than Mexican-Americans (*p* < 0.05 for each comparison). When stratified by age, no differences were observed by race/ethnicity within the 6–11 or 12–19 year age group (Fig. 3). Within the 20–39 year age group, Non-Hispanic Blacks, Non-Hispanic Whites, and Non-Hispanic Other had a higher seroprevalence than Mexican-Americans (*p* < 0.05, *p* < 0.05 and *p* < 0.001, respectively). Non-Hispanic Blacks and Non-Hispanic Other had a higher seroprevalence than Other Hispanic (*p* < 0.05 for each comparison). Within the 40–49 year age group, Non-Hispanic Blacks and Non-Hispanic Whites had a higher seroprevalence than Mexican-Americans (*p* < 0.05 for each comparison).

Among Mexican-Americans, those aged 6–11 years had a higher seroprevalence than those aged 20–39 and 40–49 years (*p* < 0.05 for each comparison). Those aged 12–19 years had a higher seroprevalence than those aged 20–39 and 40–49 years (*p* < 0.05 for each comparison).

Among Other Hispanics, those aged 6–11 years had a higher seroprevalence than those aged 20–39 and 40–49 years (*p* < 0.05 for each comparison). Those aged 12–19 years had a higher seroprevalence than those aged 20–39 and 40–49 years (*p* < 0.05 for each comparison).

No statistically significant differences were observed by age group among Non-Hispanic Blacks, Non-Hispanic Whites, or Non-Hispanic Others.

### Seroprevalence of poliovirus type 3 antibody

Poliovirus seroprevalence did not differ by gender but was higher among those aged 6–11 years compared to those aged 12–19, 20–39 and 40–49 years (*p* < 0.001 for each comparison, Table 1, Fig.1). Those aged 12–19 and 40–49 years had a higher seroprevalence than those aged 20–39 years (*p* < 0.05 and *p* < 0.001, respectively). When stratified by gender, those aged 6–11 years had a higher seroprevalence than those aged 12–19, 20–39, and 40–49 for both males and females (*p* < 0.05, *p* < 0.001, and *p* < 0.001, respectively for males and *p* < 0.001, *p* < 0.001, and *p* < 0.05, respectively for females). Those aged 40–49 years had a higher seroprevalence than those aged 12–19 and 20–39 years for females (*p* < 0.05 and *p* < 0.001, respectively) and those aged 12–19 years had a higher seroprevelance than those aged 20–39 years for males (*p* < 0.05). Among those aged 40–49 years, males had a higher seroprevalence than females (*p* < 0.05). Within the other age groups, no differences by gender were observed. No significant difference in seroprevalence was observed between U.S. born group and non-U.S. born group.

Non-Hispanic Blacks and Non-Hispanic Whites had higher seroprevalence compared to Mexican-Americans (*p* < 0.05 for each comparison). When stratified by age, for those aged 6–11 years, Mexican-Americans had a higher seroprevelance than Non-Hispanic Whites and Non-Hispanic Other (*p* < 0.05 for each comparison, Fig. 4). Within the 12–19 year age group, Other Hispanics had a higher seroprevalence than Mexican-Americans (*p* < 0.05). Within the 20–39 year age group, Non-Hispanic Blacks had a higher seroprevalence than Mexican-Americans (*p* < 0.05). Within the 40–49 year age group, Non-Hispanic Blacks, Non-Hispanic Whites, and Other Hispanics had a higher seroprevalence than Mexican Americans (*p* < 0.05 for each comparison).

Among Mexican-Americans, those aged 6–11 years had a higher seroprevalence than those aged 12–19, 20–39, and 40–49 years (*p* < 0.001 for each comparison). Among Other Hispanics, those aged 6–11 years had a higher seroprevalence than those aged 20–39 years (*p* < 0.001) and those age 12–19 years had a higher seroprevalence than those aged 20–39 years (*p* < 0.05). Among Non-Hispanic Whites, those aged 6–11 years had a higher seroprevalence than those aged 12–19, 20–39, and 40–49 years (*p* < 0.05, *p* < 0.001, and *p* < 0.05, respectively) and those age 40–49 years had a higher seroprevalence than those aged 20–39 years (*p* < 0.05). Among Non-Hispanic Blacks, those aged 6–11 years had a higher seroprevalence than those aged 12–19, 20–39, and 40–49 years (*p* < 0.001, *p* < 0.001, and *p* < 0.05, respectively). Among Non-Hispanic Other, those age 12–19 years had a higher seroprevalence than those aged 20–39 years (*p* < 0.05).

### Comparison of antibody seroprevalence between the three poliovirus serotypes

Seroprevalence for serotype 1 was higher than serotype 3 for all four age groups: 6–11, 12–19, 20–39, and 40–49 years (*p* < 0.05, *p* < 0.001, *p* < 0.001, and *p* < 0.001, respectively, Fig. 1) as well as for each gender (*p* < 0.05 for each comparison). Seroprevalence for serotype 2 was also higher than serotype 3 for all four age groups (*p* < 0.001 for all age groups) as well as for each gender (*p* < 0.001 for each comparison). Seroprevalence for serotype 2 was greater than serotype 1 for those aged 12–19 years and 20–39 years (*p* < 0.001 for both age groups). Seroprevalence for serotype 2 was greater than serotype 1 among both females and males (*p* < 0.001 and *p* < 0.05, respectively).

Seroprevalence for serotype 1 was higher than serotype 3 for all race/ethnicity groups: Mexican-American, Other Hispanic, Non-Hispanic Whites, Non-Hispanic Blacks, and Non-Hispanic Other (*p* < 0.001, *p* < 0.05, *p* < 0.001, *p* < 0.001, and *p* < 0.05, respectively). Seroprevalence for serotype 2 was also higher than serotype 3 for all race/ethnicity groups (*p* < 0.001 for groups other than Non-Hispanic Other [*p* < 0.05]). Seroprevalence for serotype 2 was greater than serotype 1 for Other Hispanic, Non-Hispanic Whites, Non-Hispanic Blacks, and Non-Hispanic Other (*p* < 0.05, *p* < 0.001, *p* < 0.001, and *p* < 0.05, respectively).

## Discussion

Overall seroprevalence for antibodies against poliovirus serotypes 1-3 was high in the US population aged 6–49 years during 2009–2010. Seroprevalence was highest among those aged 6–11 years, a group likely to have recently completed a full poliovirus vaccine schedule. A general trend of lower seroprevalence with increasing age was observed; however, differences for each age group varied by serotype. Of note, seroprevalence for serotype 3 was higher for those aged 40–49 years compared to ages 20–39 and 12–19, which is an unexpected result. It is not clear what led to this finding; it may reflect exposure to WPV serotype 3 in a proportion of this age group during childhood, but this is not known. U.S. born persons had higher seroprevalence than non-U.S. born persons for serotypes 1 and 2. Differences by race/ethnicity varied by age and serotype, and while there was a tendency for higher seroprevalence rates among non-Hispanic Blacks and Whites, this was not a universal finding. Lower seroprevalence among some subgroups may indicate certain subpopulations may be at increased risk for transmission and outbreaks if poliovirus was introduced; however, the seroprevalances among all subgroups evaluated were near or above the expected crude herd immunity threshold in a developed country, which has been estimated at a range of 80–86 % [13]. Nevertheless, given that overall seronegative rates were 6.1 %, 3.0 %, and 16.9 % for serotypes 1, 2, and 3, respectively, there remains some low level of susceptibility. As long as poliovirus circulation continues anywhere in the world, importations remain a risk and consequently, there remains a limited risk of possible outbreaks among unvaccinated subpopulations.

This study has some limitations. The analyses relied on data from NHANES 2009-10 and it is unknown if these results are generalizable to the current status of poliovirus immunity in the US. It may be that some of the differences observed are no longer present as the cohort of children and adolescents move into older age groups. In addition, NHANES did not collect poliovirus disease or vaccination history so we were unable to distinguish between immunity due to infection versus vaccination. Among US-born persons, natural infection would be expected to be very rare since circulation of WPV has been at an extremely low level (or zero) in the US during the lifetime of all but the oldest of study subjects. Acquisition of infection by a US-born person while traveling abroad is a possibility, but is also likely to be relatively rare. Finally, the proportion of individuals that were surveyed and ultimately tested varied by age group, race/ethnicity, and U.S. birth status and NHANES cannot identify small potentially susceptible subpopulations with precision.

## Conclusions

Results from NHANES 2009–2010 indicate high seroprevalence to poliovirus in the civilian non-institutionalized U.S. population aged 6–49 years and provides evidence to support prevention of sustained disease transmission in the U.S. in case of an importation. WPV remains endemic in only 2 countries, limiting the risk of importation to the U.S., but not eliminating it. Monitoring seroprevalence is an important component of understanding risks for polio in the U.S. Continued high vaccine coverage remains paramount in the near future while the world completes the global elimination of polio.

## Abbreviations

CCID_50_, cell culture infectious dose 50 %; CDC, U.S. Centers for Disease Control and Prevention; cVDPV, circulating vaccine-derived poliovirus; DVD, Division of Viral Diseases, CDC; HEp-2(C) cells, human cervix carcinoma cell line; IPV, inactivated poliovirus vaccine; NCHS, National Center for Health Statistics, CDC; NHANES, National Health and Nutrition Examination Survey; OPV, oral poliovirus vaccine; U.S., United States of America; WPV, wild poliovirus
